# Transposable elements in cancer as a by-product of stress-induced evolvability

**DOI:** 10.3389/fgene.2014.00156

**Published:** 2014-05-30

**Authors:** Tobias Mourier, Lars P. Nielsen, Anders J. Hansen, Eske Willerslev

**Affiliations:** ^1^Natural History Museum of Denmark, Centre for GeoGenetics, University of CopenhagenCopenhagen, Denmark; ^2^Department of Virology and the Danish National Biobank, Statens Serum InstitutCopenhagen, Denmark

**Keywords:** transposable elements, stress response, evolution, evolvability, cancer

## Abstract

Transposable elements (TEs) are ubiquitous in eukaryotic genomes. Barbara McClintock’s famous notion of TEs acting as controlling elements modifying the genetic response of an organism upon exposure to stressful environments has since been solidly supported in a series of model organisms. This requires the TE activity response to possess an element of specificity and be targeted toward certain parts of the genome. We propose that a similar TE response is present in human cells, and that this stress response may drive the onset of human cancers. As such, TE-driven cancers may be viewed as an evolutionary by-product of organisms’ abilities to genetically adapt to environmental stress.

## TRANSPOSABLE ELEMENTS AND GENOME EVOLUTION

Transposable elements (TEs) and their remnants constitute between half and two thirds of the human genome (Lander et al., 2001; de Koning et al., 2011), and are with few exceptions (Arkhipova and Meselson, 2000; Gardner et al., 2002) found in all sequenced eukaryotic genomes. TEs are divided according to the presence or absence of an RNA intermediate in their replication cycle (elements referred to as retroelements or DNA transposons, respectively). Retroelements are further divided into elements with or without long terminal repeat (LTR) sequences (Kazazian, 2004). The activity of DNA transposons in the human lineage ceased around 40 million years ago and now constitute 3% of the extant human genome (Lander et al., 2001; Pace and Feschotte, 2007). No LTR retroelements are considered to be actively spreading in the human genome, although the endogenous retrovirus, HERV-K is polymorphic among human populations (Belshaw et al., 2005) and capable of forming viral particles (Boller et al., 2008). In total, endogenous retroviruses constitute around 8% of the human genome. Three non-LTR retroelements are actively spreading in the human genome; the autonomous L1 LINEs and the two non-autonomous SINEs, Alu and SVA. Full-length L1 LINE elements are around 6 kilo base pairs (kbp) in size and contain two open reading frames encoding the enzymes required for transposition (Ostertag and Kazazian, 2001). More than half a million L1 sequences constitute approximately 17% of the human genome, although only around 150 elements are full-length (Penzkofer et al., 2005) and presumably even fewer – perhaps only a handful – actively transpose (Brouha et al., 2003). Alu and SVA elements are short (~300 bp and typically < 1 kbp, respectively) and do not encode any proteins (Batzer and Deininger, 2002; Wang et al., 2005). Rather, these elements rely on the enzymatic activity provided by L1 elements [established for Alu and presumed for SVA (Dewannieux et al., 2003; Ostertag et al., 2003)]. The human genome harbors more than 1.5 million Alu copies and around 500 SVA copies, covering 11% of the total genomic sequence.

## CELLULAR TE REPRESSION

The activity of TE can be repressed in several ways and at different stages of activity. Most notably, DNA or histone methylation abolishes transcription of TE sequences (Slotkin and Martienssen, 2007). The production of double-stranded RNAs (dsRNAs) from TE sequences may result in the production of small-interference RNAs (siRNAs) targeting transcribed TE sequences for degradation (Ahlquist, 2002; Yang and Kazazian, 2006), or Piwi interacting RNAs that guides *de novo* methylation during early development (Aravin et al., 2007; Carmell et al., 2007; Kuramochi-Miyagawa et al., 2008). Generation of TE dsRNA sequences may stem from transcription of both strands within specific TE sequences (Yang and Kazazian, 2006; Li et al., 2014). Alternatively, different loci could produce complementary TE RNA strands, as for example from the “passive” transcription of TEs residing in mRNA introns (Mourier, 2011).

L1, Alu, and SVA transcripts may undergo RNA editing through C-to-U deamination by members of the APOBEC3 protein family, inhibiting transposition (Schumann, 2007), and the Trex1 endonuclease metabolizes reverse-transcribed DNA from human L1 sequences and mice LTR elements in human cell cultures (Stetson et al., 2008).

The ORF1 protein from L1 is sequestered in stress granules where it co-localizes with the siRNA-processing RISC complex and closely associates with the putative RNA helicase MOV10 (Goodier et al., 2007, 2012). It is proposed that MOV10 recruits L1 ribonucleoproteins to stress granules, leading to silencing and degradation (Goodier et al., 2012). Interestingly, the MOV10 paralog MOV10L1 binds MILI and MIWI proteins that associate with piRNAs, and the knockout of MOV10L1 leads to increased L1 and LTR transcription in mice (Frost et al., 2010).

As previously pointed out, the redundancy between different TE repression mechanisms provides functional strength but also a vulnerability due to interdependence (Carreira et al., 2014). Despite the plethora of suppression mechanisms, TEs are continuing their activity in our genomes as witnessed by the level of TE polymorphisms between humans and chimpanzee (Hedges et al., 2004; Lee et al., 2007), between human individuals (Bennett et al., 2004; Wang et al., 2006; Stewart et al., 2011) and between homologous chromosomes within individuals (Levy et al., 2007; Wang et al., 2008). Importantly, human TE transcription and transposition has been recorded within somatic tissues of individuals (Belancio et al., 2010; Baillie et al., 2011).

## TEs AND GENOME EVOLUTION

If inserted into existing structures, TEs may disrupt genomic functions. However, this so-called insertional mutagenesis is by no means the only way genetic functions can be altered by TE activity. Inserted TE sequences may act as promoters driving transcription of neighboring genes, which is most prominent for LTR elements (Dunn et al., 2003, 2006) but is also observed for hypomethylated LINEs in cancer tissues (Roman-Gomez et al., 2005; Wolff et al., 2010). If inserted within transcribed genetic sequences, L1 elements may repress gene expression by inhibiting transcriptional elongation (Han et al., 2004). A survey of cap-selected human transcripts revealed that 5–15% of all transcripts from different tissues were initiated within TEs (Faulkner et al., 2009) testifying the impact TE sequences have on the total transcriptome. Ectopic recombination between non-homologous TEs leading to chromosomal changes has been inferred for all types of human TEs (Hughes and Coffin, 2001; Han et al., 2005; Sen et al., 2006). The reverse transcriptase machinery from L1 elements may occasionally insert exogenous mRNAs, resulting in the formation of processed pseudogenes (Esnault et al., 2000), of which there are around 8000 in the human genome (Zhang et al., 2003). Similarly, if the transcription of L1 elements continues into flanking sequence and genes, these chimerical transcripts may be reverse transcribed and inserted resulting in so-called sequence transduction which is estimated to constitute around 1% of the human genome (Pickeral et al., 2000). Alu elements residing in untranslated regions and introns often provide splice signals leading to the creation of novel exons (Lin et al., 2008; Keren et al., 2010), and are targets for RNA editing (Paz-Yaacov et al., 2010; Bazak et al., 2014), the level of which have implications for gene regulations (Chen et al., 2008). Furthermore, the transcriptional activity of the murine SINE B2 was shown to act as an insulator for chromatin modification between genomic domains (Lunyak et al., 2007). Notable examples of TE being recruited for genomic functions through evolution include the syncytin genes in placentas, where the envelope proteins from endogenous retroviruses promote the cell fusions between mother and fetus (Haig, 2012; Chuong, 2013), and the enhancer activity of an ancient SINE regulating neural development in mammals (Bejerano et al., 2006). Accordingly, TEs have contributed significantly to mammalian genome evolution by either creating or deleting elements, changing activity of existing elements, or shuffling genomic regions (Deininger et al., 2003; Kazazian, 2004; Mourier, 2005; Cordaux and Batzer, 2009; Chuong et al., 2013).

## CANCER AND EVOLVABILITY

**FIGURE 1:**
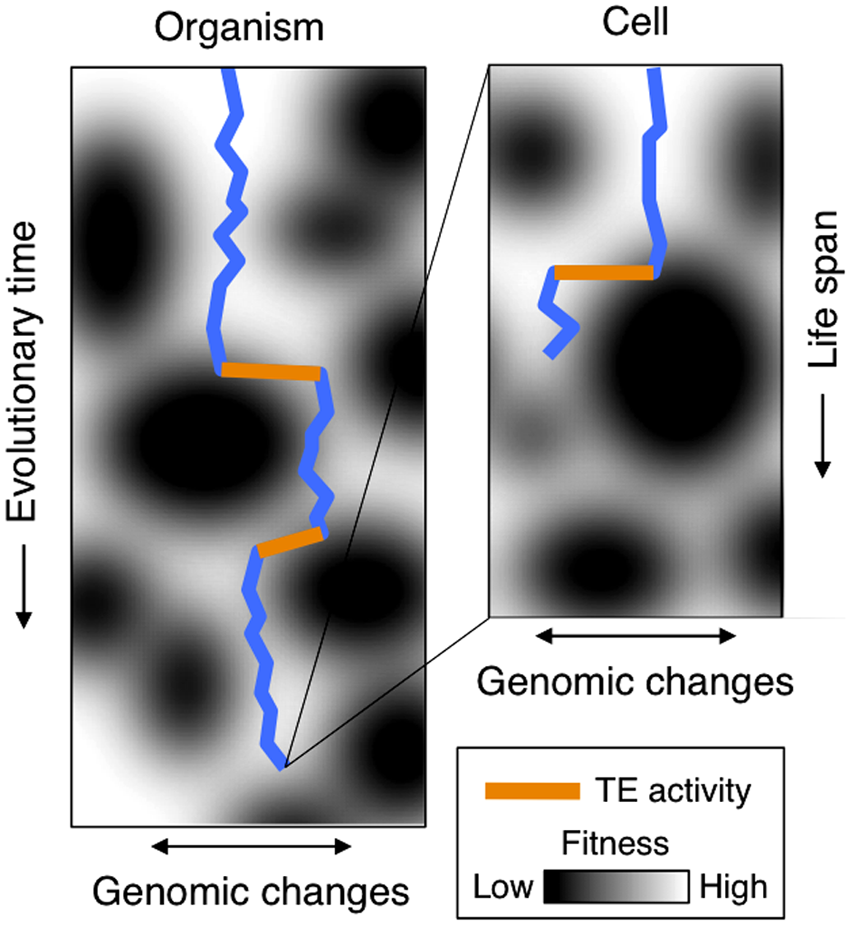
**Left: the trajectory of an organism through a fitness landscape (blue line)**. As time passes (vertical movement), genomic changes (horizontal movements) are accumulated. When encountering regions of lower fitness (dark areas), e.g., caused by chancing environments, TE may induce substantial genomic changes as indicated by orange lines. Right: the evolution within an individual cell’s life span. When encountering stress (dark areas), activation of TE induces genomic changes, which in turn may lead to cancer progression.

TE activity is readily reported in cancer cells (Asch et al., 1996; Faulkner et al., 2009; Lamprecht et al., 2010; Romanish et al., 2010; Levin and Moran, 2011; Gualtieri et al., 2013) and is commonly associated with an overall breakdown of cellular TE repression mechanisms, such as methylation (Wilson et al., 2007; Daskalos et al., 2009; Wolff et al., 2010; Xiang et al., 2010; Pavicic et al., 2012). Implicitly, this suggests that increased TE activity is a derived and in essence non-adaptive response in cancer cells. Yet, TE activity during stress may – from an evolutionary perspective – be viewed as a means by which organisms can keep up rates of genetic adaptations to changing conditions. Changing environments stress an organism and cause fixation of favorable genetic changes by natural selection. This again results in genetic adaptation of the species or cells. Here, we argue that cancer related to human TE activity can be viewed as a by-product of genome flexibility meant for effectively adapting the cell (**Figure 1**). And, importantly, that TE activity induced by external stresses should thus be regarded as an evolutionary adaptive mechanism.

Given the functional diversity of human TEs, a global activation of the entire TE population would provide a crude measure to affect the genotype. Rather, one would imagine that the probability of TEs creating adaptive genotypes increases with specificity (different activity levels for different TEs) and/or targeting (TEs are predominantly inserted in certain regions of the genome). In the following, we briefly review examples of highly targeted TE activity from non-mammalian model organisms, and end by providing observations suggesting that stress-induced TE activity in mammalian cells can be both specific as well as targeted. Hence, environmental stimuli may trigger the formation of genetic changes through TE activity at all stages of cancers, including the very onset.

## TEs AND EVOLVABILITY

All living organisms face an apparent paradox. On the one hand, their replication over time should be as exact as possible to maintain proper function, but on the other hand, organisms must evolve for their lineage to survive changing environments. When cells encounter changing environments (whether permanently or temporarily, the latter interchangeably referred to as stress) they may induce genomic changes potentially generating adaptive mutations.

When multiple genes carry out overlapping functions, individual genes are allowed to diverge from their optimum without affecting the phenotypic outcome (Hermisson and Wagner, 2004), allowing an exploration of genotype space with little non-adaptive cost (Masel and Trotter, 2010). Hence, genomes will carry in them a range of mutations (termed cryptic variation) that can be released by the activity of so-called genetic capacitors (Rutherford and Lindquist, 1998; Bergman and Siegal, 2003; Masel and Trotter, 2010). In this context, it is noteworthy that inhibition of the capacitor hsp90 deregulates the activity of TEs (Specchia et al., 2010), suggesting a role for TEs in regulators of evolvability.

Importantly, the ability to generate genetic variability, termed evolvability, does not imply foresight or teleology (Poole et al., 2003). Errors introduced by the replication machinery thus represent one factor contributing to evolvability. Compared to single base pair substitutions, inducing TE activity provides a highly efficient way of generating genetic variability. Although the vast majority of TE insertions are deleterious (Boissinot et al., 2001, 2006), they may occasionally modify existing gene structures or merely alter the expression profiles of genes (both temporally and spatially) in a way that proves adaptive to the host.

Introducing genomic changes when facing environmental stress – as suggested by the TE pioneer Barbara McClintock (McClintock, 1984) – makes intuitive sense for organisms with limited mobility, such as plants and unicellular organisms that are unable to evade suboptimal environments and are forced to adapt to the external changes. Among such organisms it is well established that cellular stresses can lead to highly specific TE activity, often minimizing deleterious insertions and maximizing the potential for adaptive regulatory changes. Below we review a few selected examples of specific TE activity in non-mammalian model organisms.

## EXAMPLES OF STRESS-INDUCED TE ACTIVITY IN MODEL ORGANISMS

Stress-induced TE activity is found across a multitude of organisms (Arnault and Dufournel, 1994; Capy et al., 2000; Morales et al., 2003; Garcia Guerreiro, 2012), and although this may reflect a genome-wide collapse of cellular TE suppression, numerous examples of specifically induced TE activity in response to stress are known. In compact genomes, TE insertions are usually highly targeted toward genomic regions where interference with endogenous functions is minimized. This is seen in fission yeast where LTR elements are preferentially inserted upstream of polymerase II transcribed genes (Bowen et al., 2003; Leem et al., 2008). Upon low oxygen levels, a specific transcription factor induces LTR transposition in fission yeast and in turn, transcription of downstream genes (Sehgal et al., 2007). A similar specific activation is found in budding yeast, where depletion of adenylic nucleotides induces a transcription factor, activating the LTR transcription (Servant et al., 2012). In the tobacco plant, *Nicotiana tabacum*, the Tnt1 retrotransposon is induced by abiotic stresses (Mhiri et al., 1997) and share transcriptional activation regions with stress-inducible genes (Grandbastien et al., 1997).

The insertion of Ty5 LTR elements in budding yeast is targeted toward heterochromatic regions through the binding between the Ty5 integrase and a heterochromatin component (Gai and Voytas, 1998). Upon exposure to cellular stress factors, integrase phosphorylation decreases which abolishes the targeting and results in insertions near genes, potentially altering their function and activity (Ebina and Levin, 2007). Hence, the result of stress is in this case not restricted to the sheer magnitude of TE activity, but rather on the potential effect TE activity may impose on the genotype. In the above cases, TE activity is a direct, inducible result of stress, and not the indirect by-product of cellular turmoil.

That the genotypic effect of TE activity that penetrates to the phenotype is illustrated in fission yeast, where heat-induced TE activity changes the expression of stress genes downstream to newly inserted TE sequences (Feng et al., 2012), leaving an immediate effect on gene functionality. Some fixed TE insertions fit well with being triggered by stress, and eventually conferring the host with a modified – and adaptive – response toward the stressor. Such examples include the soybean, *Glycine max* in which the disruption of the *gmphyA2* gene by a TE insertion is associated with high latitudes (Kanazawa et al., 2009), the mosquito, *Culex pipiens* where the disruption of the *cpm1* receptor gene by a TE insertion confers resistance to a specific toxin (Darboux et al., 2007), and *Drosophila* where a TE-mediated gene truncation increases pesticide resistance (Aminetzach et al., 2005).

## SPECIFICITY AND TARGETING OF MAMMALIAN TE ACTIVITY

If mammalian stress-induced TE activity reflects the evolvability observed in other model organisms, TE activity is expected to be non-random. First, insertions should be targeted so that certain genomic regions are preferential targets for TE insertions. Second, given the functional diversity of mammalian TEs, the response should be specific, so that different TEs are activated by different environmental stress factors. In the following, we review findings suggesting that mammalian TE-activity can be both specific and targeted.

During heat shock, mammalian cells up-regulate a specific set of genes encoding heat shock proteins while repressing a large repertoire of otherwise constitutively expressed genes (Richter et al., 2010). In mammals, the polymerase III (pol III) transcribed SINEs are up-regulated during heat shock and enter the complexes that target promoters of repressed genes (Ponicsan et al., 2010). Intriguingly, in humans and mouse two different and unrelated SINEs [the human Alu is derived from a 7SL RNA (Batzer and Deininger, 2002), the mouse B2 from a tRNA (Daniels and Deininger, 1985)] are part of this heat shock response (Allen et al., 2004; Mariner et al., 2008). Hence, different SINEs have been recruited for this task during mammalian evolution, suggesting that increased transcription during heat shock is a common feature of these SINEs. Importantly, heat shock activation is specific to SINEs as other pol III transcribed genes are unaffected (Liu et al., 1995), suggesting that increased SINE expression is not simply a result of a general increase in pol III activity.

Hunter and colleagues recently reported the silencing of TE sequences through histone methylation in the brains of stressed rats (Hunter et al., 2012). Interestingly, this revealed a surprising differentiation between TE types and brain regions, and most strikingly that histone methylation did not target L1 sequences. As transgenic L1s are in fact actively transposing in rodent hippocampus (Muotri et al., 2005; Kuwabara et al., 2009), there is no reason to suspect that L1 should be less active than other rodent TEs in this part of the brain. This differential silencing is consistent with methylation not acting as a global response to an overall increase in TE activity. Although the observed differences between TE activity levels may reflect the diversity by which different TEs are normally repressed – so that specific activation is a result of specific de-repression – it shows that an external stimulus may elicit a specific TE response rather than being restricted to a global elevation of TE activity. A recent example comes from the discovery of hypomethylation of specific human TE subfamilies in a tissue-specific manner, resulting in the gain of enhancer marks, which strongly correlated with the regulation of nearby genes (Xie et al., 2013).

One element of TE targeting comes directly from the fact that integrations are restricted to open chromatin regions. This is apparent in mammalian brains where somatic LINE insertions are enriched in the vicinity of neuronal genes (Muotri et al., 2005; Baillie et al., 2011). The finding that different environmental factors induce L1 transposition through different bHLH/PAS proteins opens the possibility of differentiated targeting of L1 insertions during different types of stresses (Ishizaka et al., 2012), and experimental findings hint that TE insertions are targeted beyond the apparent mechanistic necessity of open chromatin. Howard and colleagues created transgenic mice with a functional loss of DNA methyltransferase activity, which results in genomic hypomethylation and development of thymic lymphomas (Howard et al., 2008). In a sample of 16 transgenic mice, 7 were found to have independent IAP insertions into the introns of the Notch gene (Howard et al., 2008). Similarly, Wimmer et al. reported a genomic hotspot for TE insertions in the neurofibromin 1, NF1 gene (Wimmer et al., 2011), and more than a handful of human genomic loci are known for which multiple, independent TE insertions are reported (Chen et al., 2005; Hancks and Kazazian, 2012). Finally, it should be noted that wide variations in the rates between mobilized TEs are observed among different human cancers (Lee et al., 2012).

Interestingly, apparent TE targeting can be observed over evolutionary times, as a study on the contribution of TE sequences to human mRNA untranslated regions found genes responding to stress and external stimuli to harbor more TE sequence than other gene classes (van de Lagemaat et al., 2003). There is obviously a strong ascertainment bias in the above observations as only certain phenotypic outcomes are considered and that highly deleterious insertions are immediately pruned by natural selection. Yet, this nevertheless suggests that the genomic distribution of human TE insertions is far from random.

## CONCLUDING REMARKS

TEs typically contain several functional components that can be moved around the genome and inserted into novel genetic contexts. As different environmental stimuli may elicit differential activity of TE classes, and as insertions can be highly non-random, it is apparent that TEs provide a highly efficient mechanism for evolvability.

In line with previous speculations (Hauptmann and Schmitt, 2006), we have suggested that stress-induced TE activity driving human cancers is a reminiscent of TE-inducible evolvability (**Figure 1**). However, we here address the different evolution and ecology of eukaryotic cells in single and multicellular organisms, which is essential for this discussion.

We have highlighted examples of mammalian stress-induced TE activity being both targeted and specific. Importantly, TE activity may not necessarily be limited to transposition but could consist of transcriptional activity only. Whether the ability of stress-inducible TE activity serves as an adaptive host response within our cells or it reflects an inherent ability of TEs of being activated upon changing environments is unknown. Perhaps, the innate properties that have allowed the exaptation of TEs in cellular stress responses (such as the heat shock response, above) are the very same properties that underlie the ability to induce evolvability. This way, these properties would be selected for because of the former (heat shock) without the latter (evolvability) necessarily being adaptive. In organisms with a sequestered germ line, somatic TE insertions will not be passed on to future generations. Yet it is possible that somatic cells and tissues modify their hard-coded genetic information during an individual’s life span. The notion of the mammalian genome as a stable and inert code may largely result from our experimental inability to record changes between tissues and stages. Furthermore, somatic TE transposition may be limited to a few insertions in each cell making these hard to detect in healthy cells without the clonal amplification seen in cancer (Goodier, 2014). Although it is currently unknown just how fluid our somatic genomes are, recent technological advances have revealed mosaicisms of copy number variations within and between tissues (Abyzov et al., 2012; O’Huallachain et al., 2012) as well as extensive genetic heterogeneity in tumor cells (Aktipis and Nesse, 2013). As such, the notion of the mammalian somatic genome as a stable and inert code is questioned, and one cannot entirely rule out that certain somatic mutational profiles are adaptive for the organism as a whole.

It is evident that unleashed TE activity (e.g., caused by global aberration of genomic methylation) in neoplasm may induce further genetic changes accelerating cancer progression. However, the existence of stress-inducible TEs suggests that environmental stimuli alone may trigger the formation of genetic changes through TE activity at all stages of cancers, including the very onset. Similarly, it has recently been suggested that stress-induced TE activity may alter the neuronal genotypes in the human hippocampus, and that this could potentially be linked to neuronal disorders caused by severe stress (Hunter et al., 2013). Importantly, we do not advocate the view that TE activity underlies all human cancers. Lee and colleagues only found somatic TE activity in epithelial cancers (Lee et al., 2012), and TE involvement in cancers may be restricted to such plastic and reprogrammable tissues (Carreira et al., 2014).

The presented notion has several implications as different cellular stresses may elicit different TE responses in different cell types. Subsequently, different TE responses may underlie different cancers. This prompts for investigations not only into the genetic variability between cells in terms of TEs but also into the TE responses induced by cellular stress types. The advent of single-cell sequencing strategies (Evrony et al., 2012; Macaulay and Voet, 2014) combined with high-throughput sequencing technology makes it possible to test the above view and elucidate the involvement of TEs in cancer onset and progression. For example, exposing cell cultures to different external stresses should result in different TE responses, either in terms of TEs being activated or in the genomic regions targeted by their insertions. Similarly, careful examination of cancer types for which different stages of cancer progression is available should reveal fixed TE insertions present in the earliest stages. Hence, a combination of sequencing of stressed cells *in vitro* and of cancer cells *in vivo* could provide a rarely established link between the external environment and cancer genotypes in non-heritable cancers.

## Conflict of Interest Statement

The authors declare that the research was conducted in the absence of any commercial or financial relationships that could be construed as a potential conflict of interest.
